# Supplementary material for: Exploring experiences of work-related inequitable treatment among international medical graduates (IMGs): A sequential explanatory mixed methods study
Source: PLoS One. 2025 Feb 21;20(2):e0319230. doi: 10.1371/journal.pone.0319230 (PMC11845036; doi:10.1371/journal.pone.0319230)
Supplement: S1 Appendix — (PDF) [file pone.0319230.s001.pdf]

Survey questionnaire

Overall, when compared to a local Australian graduate, do you think that being an IMG is an advantage or disadvantage?	◯ IMGs are very disadvantaged<br>◯ IMGs are slightly disadvantaged<br>◯ IMGs are neither advantaged nor disadvantaged when compared to Australian Medical Graduates<br>◯ IMGs are slightly advantaged<br>◯ IMGs are very advantaged
Please select your reasoning for the previous question.<br><br>Select all that apply	☐ Specialty choice<br>☐ Geographical location choice<br>☐ Career progression<br>☐ Registration and/or bureaucratic requirements<br>☐ Assessment requirements<br>☐ The way patients or family treat IMGs<br>☐ The way staff treat IMGs<br>☐ Other/ none of the above
Please specify	______________________________
Since migrating, have you ever felt discriminated working/attempting to work as an IMG in Australia?<br><br>Discrimination definition: Discrimination is the unfair or prejudicial treatment of people and groups, based on characteristics they cannot change eg, race, age, gender, sexual orientation	◯ Yes<br>◯ No
In the last 5 years, have you ever felt discriminated working as an IMG in Australia?	◯ Yes<br>◯ No
Why do you think you have been discriminated against whilst working/attempting to work in Australia in the last 5 years?<br>Please select all that apply	☐ gender<br>☐ race or skin colour<br>☐ culture<br>☐ language/accent<br>☐ religion<br>☐ name<br>☐ IMG status (ie, holding a foreign degree)<br>☐ immigration status or nationality<br>☐ sexual orientation<br>☐ age<br>☐ disability<br>☐ marital status<br>☐ other<br>☐ not applicable- I have not experienced discrimination in Australia in the last 5 years
Please specify	______________________________
Who has discriminated against you, whilst working/attempting to work in Australia in the last 5 years?<br><br>Please select all that apply	☐ institutions or organisations; 'the system'<br>☐ senior staff members/bosses<br>☐ medical colleagues<br>☐ nurses or allied health staff<br>☐ other staff eg, administration, security officers etc.<br>☐ patients or their families<br>☐ other<br>☐ not applicable- I have not experienced discrimination in Australia in the last 5 years
Please specify	______________________________
In the last 5 years, in your opinion, has workplace discrimination in Australia affected your career progression or attainment of higher work positions?	◯ yes<br>◯ no<br>◯ Unsure<br>◯ not applicable- I have not experienced workplace discrimination in the last 5 years
In the last 5 years, in your opinion, has workplace discrimination in Australia affected your physical health?	◯ Yes<br>◯ No<br>◯ Unsure<br>◯ not applicable- I have not experienced workplace discrimination in the last 5 years
In the last 5 years, in your opinion, has workplace discrimination in Australia affected your wellbeing or mental health?	◯ Yes<br>◯ No<br>◯ Unsure<br>◯ not applicable- I have not experienced workplace discrimination in the last 5 years

The following questions relate to experiences of DISCRIMINATION or BIAS (approx. 5 mins). Please consider how often these events have happened to you in the last 5 years.

Whilst working/attempting work as a doctor in Australia in the last 5 years...

	never	a little/ rarely	sometimes/ a moderate amount	often/ frequently
I have experienced derogatory comments, gestures or teasing	○	○	○	○
I have been given an inappropriate nickname	○	○	○	○
Patients have refused my care	○	○	○	○
I have been told to 'go home to my own country' or similar	○	○	○	○
I have been unfairly subjected to complaints	○	○	○	○

Whilst working/attempting to work as a doctor in Australia in the last 5 years, ...

	never	a little/rarely	sometimes/ moderately	often/ frequently
I have felt excluded or isolated at work	○	○	○	○
People have made assumptions about my performance	○	○	○	○
I have been treated as less intelligent, or inferior	○	○	○	○
I have been treated with suspicion or rudely	○	○	○	○

Please rate the statements below, from your personal experience of being an IMG in Australia, in the last 5 years

	Strongly disagree	Slightly disagree	Neutral	Slightly agree	Strongly agree
My professional experience/ background or qualifications have been challenged or questioned	○	○	○	○	○
When compared to an Australian graduate, I need to work 'double hard' to prove myself at work	○	○	○	○	○
I have more professional experience than the job/rotation I have been allocated	○	○	○	○	○
Colleagues with less experience have progressed further than me	○	○	○	○	○
Others have unfairly taken credit/benefited from my work efforts	○	○	○	○	○
I have been expected to do work which is not related to my role	○	○	○	○	○
I have had difficulty getting job interviews	○	○	○	○	○
I have been overlooked for leadership roles	○	○	○	○	○
My professional opinion is not sought or is overlooked in a group discussion	○	○	○	○	○

	Strongly disagree	Disagree	Neutral	Agree	Strongly agree
I have limited choice about the geographical location of my work	O	O	O	O	O
I have limited choice about which specialty to undertake	O	O	O	O	O
I have limited opportunities in training	O	O	O	O	O
I have limited opportunities in job acquisition	O	O	O	O	O
I have limited opportunities in career progression	O	O	O	O	O
I have limited opportunities in professional development	O	O	O	O	O
I am not paid fairly for my work or level of experience	O	O	O	O	O
I am becoming deskilled by working in Australia	O	O	O	O	O
I have been unfairly allocated shifts or rotations that no one else wants to do	O	O	O	O	O
I have had difficulty finding	O	O	O	O	O

<u>Interview questionnaire</u>

	**INTERVIEW QUESTIONS FOR IMG PARTICIPANTS (individual interviews: qualitative study)**
2a)	What has it been like for you in Australia, being an IMG? (Probe for positive and negative experiences)
2b)	Do you think that you are treated differently to an Australian graduate? If yes, why do you think that is? (Probe for: examples of being treated better or worse, discrimination experiences, relationship with staff/ patients/ system/institution; colour, accent etc; differences with recruitment, assessments, career progression, research and academia, leadership roles, awards and professionalism
3.	What impact has being an IMG had on you [refer to above answers]? (Probe for: career change, social or personal impacts, mental and physical health)
