# Supplementary material for: Exploring experiences of work-related inequitable treatment among international medical graduates (IMGs): A sequential explanatory mixed methods study
Source: PLoS One. 2025 Feb 21;20(2):e0319230. doi: 10.1371/journal.pone.0319230 (PMC11845036; doi:10.1371/journal.pone.0319230)
Supplement: S2 Checklist — (PDF) [file pone.0319230.s002.pdf]

Good Reporting of a Mixed Methods Study checklist

O'Cathain A, Murphy E, Nicholl J. The quality of mixed methods studies in health services research. J Health Serv Res Policy. 2008;13: 92-98.

Guideline	Relevant section in article
1. Describe the justification for using a mixed methods approach to the research question.	Methods- Study design and setting
2. Describe the design in terms of the purpose, priority and sequence of methods.	Methods- Study design and setting; Data tools
3. Describe each method in terms of sampling, data collection and analysis.	Methods-participants, recruitment and sample size; Data collection<br><br>Data analysis
4. Describe where integration has occurred, how it has occurred and who has participated in it.	Data analysis – Triangulation of data from quantitative and qualitative sources<br><br>Results- Triangulation of quantitative and qualitative data
5. Describe any limitation of one method associated with the present of the other method.	Discussion- Strengths and limitations
6. Describe any insights gained from mixing or integrating methods.	Discussion- paragraphs 1&2; Strengths and limitations
