# Supplementary material for: Exploring experiences of work-related inequitable treatment among international medical graduates (IMGs): A sequential explanatory mixed methods study
Source: PLoS One. 2025 Feb 21;20(2):e0319230. doi: 10.1371/journal.pone.0319230 (PMC11845036; doi:10.1371/journal.pone.0319230)
Supplement: S3 Table — (PDF) [file pone.0319230.s003.pdf]

Demographics of survey and interview participants

		Survey participants	*Interview participants*
**Age**	Range of years	24-78	27-60
	Mean (years)	40.4	40.7
		Number of participants (%)	***Number of participants (%)***
**Gender**	Male	71/252 (28.2%)	9/36 (25%)
	Female	179/252 (71%)	27/36 (75%)
	Other	2/252 (0.8%)	0
	Total	***252***	***36***
**Marital status**	Married/de-facto	197/251 (78.5%)	28/36 (77.8%)
	Not in a married or de-facto relationship, or preferred not to say	54/251 (21.5%)	8/36 (22.2%)
	Total	***251***	***36***
**Ethnicity**	Indian	68/243 (28%)	No data
	Asian	67/243 (27.6%)	
	British/Irish	28/243 (11.5%)	
	European	23/243 (9.5%)	
	Middle Eastern	20/243 (8.2%)	
	Hispanic/ Latino	9/243	
	African/Caribbean	8/243	
	Eastern European	4/243	
	Polynesian	1/243	
	Mixed heritage	10/243 (4.1%)	
	Prefer not to say/ other	5/243	
	Total	***243***	
**TRAINING OUTSIDE AUSTRALIA**			
**Most common country of primary medical qualification (PMQ)**		1. India 45/230 (19.6%)<br>2. UK 33/230 (14.4%)<br>3. China 15/230 (6.5%)<br>4. Ireland 14/230 (6.1%)<br>5. Others 123/230 (53.5%)	1. India 9/36 (25%)<br>2. UK 7/36 (19.4%)<br>3. Egypt 3/36 (8.3%)<br>4. South Africa 3/36 (8.3%)<br>5. Others 14/36 (38.9%)
**Completed or partially completed post-**	yes	138/236 (58.5%)	23/36 (63.9%)
	no	98/236 (41.5%)	13/36 (36.1%)

**graduate training overseas**			
	Total	***236***	
**SINCE ARRIVAL IN AUSTRALIA**			
**Year of migration to Australia [range]**		1972-2023	1998-2024
**Current location of work (or residence)***	New South Wales	116/223 (52%)	20/36 (55.6%)
	Victoria	42/223 (18.8%)	6/36 (16.7%)
	Queensland	28/223 (12.6%)	5/36 (13.9%)
	Western Australia	21/223 (9.4%)	2/36 (5.6%)
	South Australia	13/223 (5.8%)	0
	Tasmania	12/223 (5.4%)	2/36 (5.6%)
	Northern Territory	4/223 (1.8%)	0
	Australian capital territory	3/223 (1.3%)	1/36 (2.8%)
	Mixed- i.e. working across	**Survey data includes 10 participants working across ≥2 states or territories*	0
**Current region of work****	Metropolitan	152/213 (71.4%)	22/36 (61.1%)
	Rural	65/213 (30.5%)	10/36 (27.8%)
	Remote	7/213 (3.3%)	
	Mixed- e.g. metropolitan and rural; or regional and remote	***Survey data Includes 11 survey participants working across mixed regions*	4/36 (11.1%)
**Current clinical employment level in Australia**	Consultant (GP or specialist)	74/190 (39%)	13/36 (36.1%)
	Registrar or Fellow or CMO	55/190 (29%)	13/36 (36.1%)
	Resident Medical Officer or Intern	52/190 (27.4%)	9/36 (25%)
	Other/ prefer not to say	9/190	1/36 (2.8%) (unemployed)
	Total	***190***	***36***
**Non-clinical jobs**	Education	9/24 (54.1%)	2/36 (5.6%)
	Research	4/24 (16.7%)	
	Medical Administration	4/24 (16.7%)	
	Others	7/24 (29.2%)	
	Total	***24***	
	yes	49/234 (20.9%)	9/36 (25%)

**Currently undertaking training**	no	181/234 (77.4%)	27/36 (75%)
	prefer not to say	4/234 (1.7%)	0
	Total	***234***	***36***
**Employment status**	Full-time	151/234 (64.5%)	No data
	Part-time	56/234 (23.9%)	
	Not working (e.g. unemployed, voluntary work, on extended leave, retired)	22/234 (9.4%)	1/36 (2.8%)
	Other/prefer not to say	5/234	
	Total	***234***	***N/A***
