# Supplementary material for: Exploring experiences of work-related inequitable treatment among international medical graduates (IMGs): A sequential explanatory mixed methods study
Source: PLoS One. 2025 Feb 21;20(2):e0319230. doi: 10.1371/journal.pone.0319230 (PMC11845036; doi:10.1371/journal.pone.0319230)

Perpetrating sources of discrimination within the last five years, as reported by affected respondents in sample

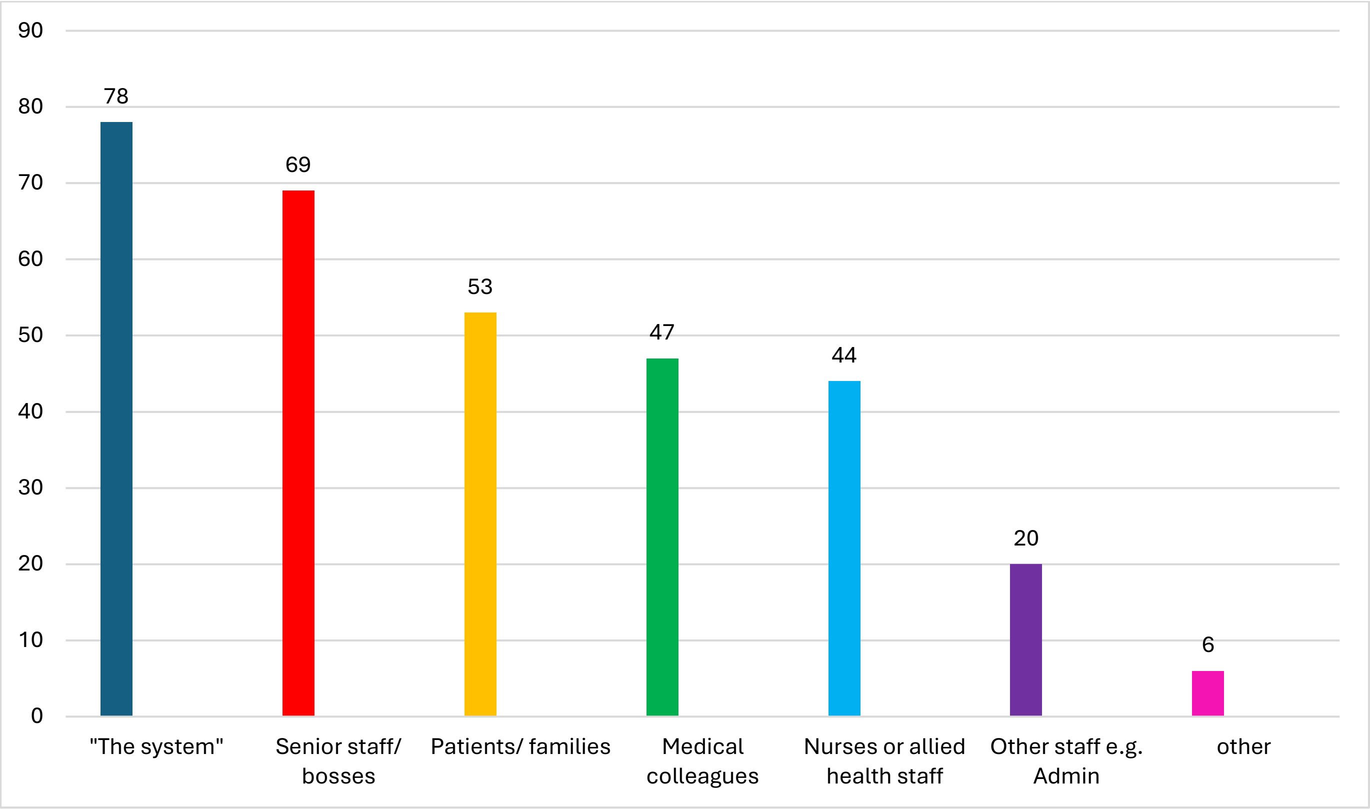

Supplement: S5 Figure — (PDF) [file pone.0319230.s005.pdf]
