# Supplementary material for: Exploring experiences of work-related inequitable treatment among international medical graduates (IMGs): A sequential explanatory mixed methods study
Source: PLoS One. 2025 Feb 21;20(2):e0319230. doi: 10.1371/journal.pone.0319230 (PMC11845036; doi:10.1371/journal.pone.0319230)
Supplement: S6 Table — (PDF) [file pone.0319230.s006.pdf]

1. REPORTS OF EXPLICIT AND SUBTLE EXPERIENCES OF DISCRIMINATION							
	**Never**<br><br>**(0)**	**A little/ Rarely**<br><br>**(1)**	**Some-times/a moderate amount**<br>**(2)**	**Often/ Frequently**<br><br>**(3)**	**Dichot. No**<br><br>**(0)**	**Dichot. Yes**<br><br>**(1,2 or 3)**	**Total**<br><br>**(n)**
1a) EXPLICIT EXPERIENCES							
I have experienced derogatory comments, gestures or teasing	18 (15%)	49 (40.8%)	44 (36.7%)	9 (7.5%)	18/120 (15%)	102/120 (85%)	120
I have been given an inappropriate nickname	85 (71.4%)	26 (21.9%)	5 (4.2%)	3 (2.5%)	85/119 (71.4%)	34/119 (28.6%)	119
Patients have refused my care	72 (61.0%)	34 (28.8%)	11 (9.3%)	1 (0.9%)	72/118 (61.0%)	46/118 (39.0%)	118
I have been told to 'go home to my own country' or similar	70 (59.3%)	31 (26.3%)	11 (9.3%)	6 (5.1%)	70 /118 (59.3%)	48/118 (40.7%)	118
I have been unfairly subjected to complaints	72 (60.5%)	24 (20.2%)	17 (14.3%)	6 (5.0%)	72/119 (60.5%)	47/119 (39.5%)	119
1b) SUBTLE EXPERIENCES							
I have felt excluded or isolated at work	21 (17.7%)	43 (36.1%)	35 (29.4%)	20 (16.8%)	21/119 (17.7%)	98/119 (82.4%)	119
People have made assumptions about my performance	14 (11.8%)	36 (30.3%)	46 (38.7%)	23 (19.3%)	14/119 (11.8%)	105/119 (88.2%)	119
I have been treated as less intelligent, or inferior	20 (17.0%)	38 (32.2%)	40 (33.9%)	20 (17%)	20/118 (17.0%)	98/118 (83.1%)	118

I have been treated with suspicion or rudely	26 (22.0%)	48 (40.7%)	29 (24.6%)	15 (12.7%)	26/118 (22.0%)	92/118 (78.0%)	118

2. REPORTS OF OTHER WORKPLACE EXPERIENCES OF DISCRIMINATION

	Strongly disagree	Slightly disagree	Neutral	Slightly agree	Strongly agree	TOTAL (n)
2a) PROFESSIONAL EXPERIENCES						
My professional experience/ background or qualifications have been challenged or questioned	11 (9.3%)	9 (7.6%)	23 (19.5%)	45 (38.1%)	30 (25.4%)	118
	**Disagree n=20/118** (16.9%)			**Agree n=75/118** (63.5%)		
When compared to an Australian graduate, I need to work 'double hard' to prove myself at work	4 (3.4%)	4 (3.4%)	12 (10.2%)	35 (29.7%)	63 (53.4%)	118
	**Disagree n= 8/118** (6.8%)			**Agree n= 98/118** (83.1%)		
I have more professional experience than the job/ rotation I have been allocated	9 (7.7%)	6 (5.1%)	37 (31.6%)	28 (23.9%)	37 (31.6%)	117
	**Disagree n=15/117** (12.8%)			**Agree n=65/117** (55.6%)		
Colleagues with less experience have progressed further than me	9 (7.7%)	4 (3.4%)	21 (18.0%)	29 (24.8%)	54 (46.2%)	117

	**Disagree n=13/117** (11.1%)			**Agree n=83/117** (70.9%)		
Others have unfairly taken credit/ benefited from my work efforts	17 (14.4%)	10 (8.5%)	33 (28.0%)	34 (28.8%)	24 (20.3%)	118
	**Disagree n=27/118** (22.9%)			**Agree n=58/118** (49.2%)		
I have been expected to do work which is not related to my role	18 (15.4%)	13 (11.1%)	40 (34.2%)	24 (20.5%)	22 (18.8%)	117
	**Disagree n=31/117** (26.5%)			**Agree n= 46/117** (39.3%)		
I have had difficulty getting job interviews	11 (9.5%)	13 (11.2%)	25 (21.6%)	24 (20.7%)	43 (37.1%)	116
	**Disagree n=24/116** (20.7%)			**Agree n=67/116** (57.8%)		
I have been overlooked for leadership roles	13 (11.1%)	5 (4.3%)	43 (36.8%)	26 (22.2%)	30 (25.6%)	117
	**Disagree n= 18/117** (15.4%)			**Agree n= 56/117** (47.9%)		
My professional opinion is not sought or is overlooked in a group discussion	11 (9.4%)	8 (6.8%)	47 (40.2%)	29 (24.8%)	22 (18.8%)	117
	**Disagree n=19/117** (16.2%)			**Agree n= 51/117** (43.6%)		

2b) SYSTEM EXPERIENCES						
I have limited choice about the geographical location of my work	8 (6.8%)	7 (6.0%)	19 (16.1%)	29 (24.6%)	55 (46.6%)	118
	**Disagree n= 15/118** (12.7%)			**Agree n= 84/118** (71.2%)		
I have limited choice about which specialty to undertake	12 (10.3%)	6 (5.2%)	32 (27.6%)	25 (21.6%)	41 (35.3%)	116
	**Disagree n= 18/118 (15.3%)**			**Agree n=66/116 (56.9%)**		
I have limited opportunities in training	10 (8.7%)	6 (5.2%)	28 (24.4%)	26 (22.6%)	45 (39.1%)	115
	**Disagree n= 16/115 (13.9%)**			**Agree n= 71/115 (61.7%)**		
I have limited opportunities in job acquisition	12 (10.2%)	8 (6.8%)	16 (13.6%)	32 (27.1%)	50 (42.4%)	118
	**Disagree n= 20/118 (16.9%)**			**Agree n= 82/118 (69.5%)**		
I have limited opportunities in career progression	9 (7.7%)	6 (5.1%)	25 (21.4%)	36 (30.8%)	41 (35.0%)	117
	**Disagree n= 15/117 (12.8%)**			**Agree n= 77/117 (65.8%)**		
I have limited opportunities in professional development	12 (10.2%)	11 (9.3%)	34 (28.8%)	29 (24.6%)	32 (27.1%)	118

	**Disagree n= 23/118 (19.5%)**			**Agree n= 61/118 (51.7%)**		
I am not paid fairly for my work or level of experience	20 (17.1%)	12 (10.3%)	30 (25.6%)	30 (25.6%)	25 (21.4%)	117
	**Disagree n= 32/117 (27.4%)**			**Agree n= 55/117 (47.0%)**		
I am becoming deskilled by working in Australia	27 (23.3%)	22 (19.0%)	21 (18.1%)	18 (15.5%)	28 (24.1%)	116
	**Disagree n= 49/116 (42.2%)**			**Agree n= 46/116 (39.7%)**		
I have been unfairly allocated shifts or rotations that no one else wants to do	14 (12.2%)	22 (19.3%)	32 (28.1%)	24 (21.1%)	22 (19.3%)	114
	**Disagree n= 36/114 (31.6%)**			**Agree n= 46/114 (40.4%)**		
I have had difficulty finding mentors	13 (11.4%)	17 (14.9%)	31 (27.9%)	30 (26.3%)	23 (20.2%)	114
	**Disagree n= 30/114 (26.3%)**			**Agree n= 53/114 (46.5%)**		
