# Supplementary material for: Exploring experiences of work-related inequitable treatment among international medical graduates (IMGs): A sequential explanatory mixed methods study
Source: PLoS One. 2025 Feb 21;20(2):e0319230. doi: 10.1371/journal.pone.0319230 (PMC11845036; doi:10.1371/journal.pone.0319230)
Supplement: S7 Figure — (PDF) [file pone.0319230.s007.pdf]

Self-reported effects of discrimination in the last five years

"In the last 5 years, in your opinion, has workplace discrimination affected your....?"

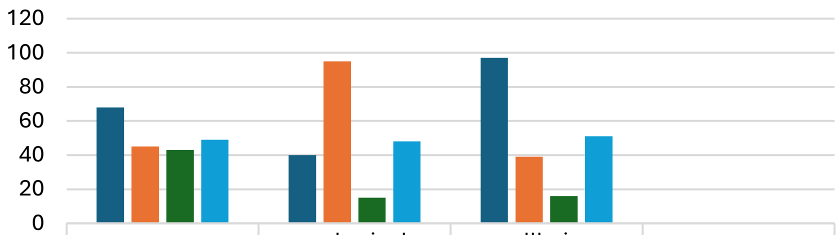

	...career progression	...physical health	...wellbeing or mental health
yes	68	40	97
no	45	95	39
unsure	43	15	16
N/A no discrimination experienced	49	48	51

yes · no · unsure · N/A no discrimination experienced
