# Supplementary material for: Exploring experiences of work-related inequitable treatment among international medical graduates (IMGs): A sequential explanatory mixed methods study
Source: PLoS One. 2025 Feb 21;20(2):e0319230. doi: 10.1371/journal.pone.0319230 (PMC11845036; doi:10.1371/journal.pone.0319230)
Supplement: S8 Table — (PDF) [file pone.0319230.s008.pdf]

Joint display of quantitative and qualitative data

Survey quantitative data	Qualitative data- survey open responses and/or interviews
Disadvantage as a construct	
The majority of respondents (181/208; 87.0%) reported that IMGs are very (103/208; 49.5%) or slightly (78/208; 37.5%) disadvantaged when compared to local graduates.<br><br>The highest reason for respondents to report disadvantage was registration/ bureaucratic requirements (125/181; 69.1%), followed by the way staff treat IMGs (88/181; 48.6%)	"…people from Indian culture especially females, tend to be submissive, because that's been something that has been drilled into your system, for about thirty years or so……In Australia, you need to talk a lot… thirty years you're told not to talk and to be quiet…and then in 2 years' time, you can't be expecting me to be chatting continuously- it takes some time." [Interviewee #10; PMQ: India]<br><br>"I mean I'm not too sure how that training program stuff works but I think there tends to be a lot of hiring of local graduates and local doctors…they seem to get more…because they have had time to build up those relationships." [Interviewee #1; PMQ: UK]
Biases- prejudice, stereotypes, discrimination	
133/208 (63.9%) respondents reported that they had felt discriminated at some point, when working/attempting to work as an IMG in Australia	**Biases toward Australian graduates**<br><br>"I think in Australia, there seems to be more nepotism in training places, compared to the UK." [Interviewee #1; PMQ: UK]

121/204 (59.3%) reported experiencing discrimination working as an IMG in Australia when asked specifically about their experiences in the last 5 years.<br><br>High rate of subtle discriminatory experiences were reported by IMGs: >75%<br><br>Statistically significant results identified when testing association of discrimination in the last five years against English as a native language, ethnicity, country of PMQ and employment status.	"And an Australian, especially a local graduate has better chances, all the time. So, it doesn't matter which level you come in, they will always prefer a local graduate, and local raised and known, and you know…to someone who comes from outside." [Interviewee #31; PMQ: Germany]<br><br>"…we [IMGs] were the ones sent out to all the far farmed places, whereas the Australian medical graduates were kept in [main city] and were nurtured and encouraged to progress and move forward and finish training." [Interviewee #32; PMQ: Trinidad and Tobago]<br><br>"It depends on what type of IMG. I feel that Caucasian candidates with excellent English are not disadvantaged. IMGs of colour and with an accent have a far harder time." [open ended survey response]<br><br>"There's a suspicion that overseas degrees aren't as good as Australian (I'm UK trained) and it's very parochial... the Australian grads use how the IMGs are treated to their advantage and advertise themselves as 'Australian graduates' and 'Australian trained' as it's seen as an advantage." [open ended survey response]<br><br>**Biases toward or against certain IMG groups**<br>"…the perception of Australians is that UK is better, but the rest of the world is sh*t… which is rubbish, yeah? They never even look at anything that doesn't speak English, yeah? While there's tonnes of countries that have a better health system than Australia." [Interviewee #31; PMQ: Germany]<br><br>"The racism and the negative perception of IMGs from Asia and Middle Eastern countries is rife. And I often find it quite awkward when [staff or patients] are discussing how they don't like such a such a doctor who's an IMG.… and sometimes I'll say, 'I'm an IMG too!' And, you know, the look on their face … and they sort of backtrack, and they'll be like 'oh no, not UK IMG!'," [Interviewee #04; PMQ: UK].<br><br>"The process is highly biased and stigmatizing towards non-UK trained doctors. I am required to sit for exams when I have more experience and training than the UK graduates." [open ended survey response]<br><br>"[As a UK graduate], I have been successful at progressing through training in major centres … this is very different from most IMGs [who] can struggle to get onto training programs due to the lack of transparency around applicant scoring and high degree of nepotism in certain specialties." [open ended survey response]

	"It can be very disheartening for IMGs like me because I see other IMGs that are UK grad get general registration very easily (some not even as competent)." [open ended survey response]<br><br>"So, the problem when I make a mistake, I find ten knives in my neck. …when the white trainee does this 'oh we come here to support you, that's fine, how can we improve this' … We don't get that. .. Hence, you have to work harder. You have to do less mistakes, more work, to please them…" [Interviewee #20; PMQ: Egypt]<br><br>"I've been treated like I'm substandard by colleagues and patients who are racist and think they're not 'seeing one of them foreign trained doctors' whom they deem as inferior." [open ended survey response]<br><br>"The public perception is a big issue too…the attitude I received on the phone was horrible. I am bilingual and naturally fluent in English, I just don't sound 'Aussie' and that was enough to trigger them [callers]." [open ended survey response]<br><br>"… people referring you as 'you're from the third world, you're not in the third world anymore'. Ah, everyone surprised with my proficiency of English. Which I must say, is at a higher level than most Australians. And the perception that I'm from the third world. And all I deserve to do here, are the sh*t jobs that no one else wants to do." [Interviewee #32; PMQ: Trinidad and Tobago]
Workplace bullying as a special manifestation of bias	
	"And it's very interesting that they would reassume that hierarchy here....I have seen the very people who talk very nicely to your Caucasian registrars, you know, snap their fingers and speak in a completely different way to the IMGs in training." [Interviewee #21; PMQ: UK]<br><br>"Our mistakes were not forgiven…we were shouted out…And I was treated so badly to the degree that I decided to resign." [Interviewee #03; PMQ: Sudan]<br><br>"That's the worst part…The person who did to me, she's an IMG herself!" [Interviewee #09; PMQ: India]

	"…the big bosses, many of them have preconceived ideas that IMGs are not good enough. I've seen locals make big mistakes and told it's all a learning process. I've seen IMGs make mistakes and get reported, put down and gossiped about." [open ended survey response]<br><br>"Any person who comes from… especially the Indian subcontinent, and the Middle East…is going to be more intimidated and more submissive than other people." [Interviewee #10; PMQ: India]<br><br>"There's a difference in the way they [*staff or patients*] would speak to someone like…one of the coloured doctors compared to like - a white doctor. It's just a way that they speak- like a manner of disrespect. It's just like a rudeness. Yeah, you can immediately tell. It's just a way, their body language. They just dismissive of them. They treat them badly." [Interviewee #27; PMQ: South Africa]<br><br>"...countries from our side, Sri Lanka, Pakistan, Bangladesh…they all know that how…they can always treat junior doctors like that. Like at home, people can sometimes be...be like rude, or can be authoritative or anything. And you will just have to go with it, because you don't have any option. So you kind of learn to live with it." [Interviewee #09; PMQ: India]
The role of systems and institutions in perpetuating discrimination	
Institutions/organisations ("the system") (78/140; 55.7%) was the most commonly reported perpetrating source of discrimination.<br><br>High rate (>70%) of IMGs reporting discrimination reported limited choice about the geographical location of own work	"I literally had to get somebody to sign a form…[Yet] somebody else, arguably more qualified, arguably more experienced, has to do like…spirometry testing, and then fail that question and have to apply to do the AMC again …it doesn't sound equitable to me." [Interviewee #12; PMQ: UK]<br><br>"… working doctors are very much seen as the cash cows for the AMC…"[open ended survey response]<br><br>"… this IMG position is … usually in hospitals where …there's a shortage of workforce…so they want to fill that position. They don't think anyone in the local community will take it. So, it's kind of disadvantaged position because … for your training, for your assessments for your exams, you really need to be in a tertiary hospital- so you will never get that [with this position]." [Interviewee #10; PMQ: India]<br><br>"I had a really difficult year. I got the worst terms ever. I got like…9 months of nights/relieving. And then I got seconded to [rural hospital]…I think being an IMG at first, you do get allocated….sort of the bottom rung of

Almost half (47%) of IMGs reporting discrimination reported not being paid fairly for their work or level of experience	rotations, because people think the good things should be given to local trainees.” [Interviewee #04; PMQ: UK].<br><br>“…you’re pretty useless after Nights. It doesn’t help you anyway with your training as well. You know, for exams, you need to have study groups, you need to attend classes….which you are not in a state to go, because you are doing Nights, or Weekends. So that was disadvantageous. Very disadvantageous.” [Interviewee #10; PMQ: India]<br><br>“I wouldn’t expect to be paid as a PGY7, but no mechanism existed for me to have my experience assessed and realise that they’re [institution] actually getting someone who’s got all this experience- ….Noone at the hospital has thought- ‘we actually have a well-experienced person that we should be trying to retain’ … from an HR point of view… they don’t really see what they are getting …for this very cheap package that I am, really.” [Interviewee #36; PMQ: South Africa]<br><br>“…he has all the responsibility of registrar, his position is registrar, so we are calling him because he is registrar- *but* he’s paid as an intern. And he cannot complain or say anything, because he needs them for [extending] the visa.” [Interviewee #19; PMQ: Italy]<br><br>“I have seen people who are the sole winner of the family, like when the income depends on this job… you’re going to just suck it [*bullying experience*] up, and then move on. So, it’s difficult.” [Interviewee #10; PMQ: India]<br><br>“I had to sell my soul for permanent residency with two years of night shifts.” [Interviewee #13; PMQ: UK]<br><br>“...IMG'S [are] at the mercies of other professional for Visa and AHPRA progression. We are held to ransom and our wages are stolen before the employer holds the aces regarding immigration & AHPRA progression and you often can't complain for fear of the above.” [open ended survey response]<br><br>“I’ve been in jobs where they seek to exploit you because of the moratorium as they know it's hard for you to get a provider number and change jobs.” [open ended survey response]

Effects of discrimination on health and career	
68/205 (33.2%) respondents reported that workplace discrimination in the last five years had affected their career progression or attainment of higher work positions.<br><br>40/198 (20.2%) respondents reported effects on their physical health<br><br>97/203 (47.8%) respondents reported effects on their mental health or wellbeing.	"The AMC through to the clinical exam has been extremely tough. But not only tough for the study, more for the psychological part, like the pressure that they put with the exam is terrible. I had post-traumatic stress disorder from that. The way they treat you during the test is horrible." [Interviewee #19; PMQ: Italy]<br><br>"AMC is one of the…as I say, horrendous exams and I know I can count on my fingers how many people I actually know have been in depression, and who has been on antidepressant treatment, just because of the amount of financial pressure and the amount of pressure this exam gives them…like in terms of passing." [Interviewee #09; PMQ: India]<br><br>"…I started getting palpitations. … And I gained a lot of weight, because there was a lot of stress eating. So, I'm still struggling to lose that weight now…yeah, once you put it on, it never goes back….so, in that way. I had difficulty with sleep, because I really don't sleep well during the day, and then usually by the end of a 3, 4-day night shift, it would take me another 3 days to come back to normal. Yeah. Lots of interruptions with that. Not a happy time. I had about 2 years with that ..." [Interviewee #10; PMQ: India]
