# Supplementary material for: Exploring experiences of work-related inequitable treatment among international medical graduates (IMGs): A sequential explanatory mixed methods study
Source: PLoS One. 2025 Feb 21;20(2):e0319230. doi: 10.1371/journal.pone.0319230 (PMC11845036; doi:10.1371/journal.pone.0319230)
Supplement: Attachment — Submitted filename: Response to reviewers.pdf [file pone.0319230.s010.pdf]

RESPONSE TO REVIEWERS

Dear Editor and reviewers,

Many thanks for your time and efforts with this manuscript: "Exploring experiences of work-related inequitable treatment among international medical graduates (IMGs): a sequential explanatory mixed methods study".

Thank you for the opportunity to revise the manuscript. Below is a step-by-step address of comments corresponded by PLOS ONE to the authors on 8th November 2024. Additions are described or highlighted below, with relevant line numbers in the tracked version; retractions are indicated with a strike-through. A revised version which fulfils the publication criteria for PLOS ONE is submitted alongside this document. Thank you again for your further consideration of the revised manuscript.

Kind regards, Dr S.J. Rebecca Healey and co-authors.

Comment or Request by Editor/Reviewer	Response from authors
**Academic Editor: Journal requirements**	
1. Please ensure that your manuscript meets PLOS ONE's style requirements, including those for file naming. The PLOS ONE style templates can be found at https://journals.plos.org/plosone/s/file?id=wjVg/PLOSOne_formatting_sample_main_body.pdf and https://journals.plos.org/plosone/s/file?id=ba62/PLOSOne_formatting_sample_title_authors_affiliations.pdf	Thank you for this reminder.<br>• Level 1,2 and 3 headings have been re-formatted throughout the document, as per PLOS ONE's style requirements<br>• File names of uploaded supplementary files have been renamed, e.g., "S4_Figure" now reads "S4_Fig.pdf" etc. Labelling of Supplemental material in the main body has been amended e.g., "S4 Figure" now reads "S4 Fig" etc.<br>• Adjustments have been made to the detail and reformatting of author affiliations in the manuscript.
2. Thank you for stating the following in the Competing Interests section: SJRH and BRN are both IMGs and work closely with IMGs, within the Workplace Based Assessment Program, Hunter New England Health. Other authors (KF, BMA, LL) do not demonstrate any competing interests.	Thank you for this advice. We can confirm that the competing interests stated do not alter our adherence to all PLOS ONE policies on sharing data and materials. The Competing Interests statement will now include the addition of "This does not alter our adherence to PLOS ONE policies on sharing data and materials", as noted in the cover letter. Thank you for

Please confirm that this does not alter your adherence to all PLOS ONE policies on sharing data and materials, by including the following statement: ""This does not alter our adherence to PLOS ONE policies on sharing data and materials.” (as detailed online in our guide for authors http://journals.plos.org/plosone/s/competing-interests).<br><br>Please include your updated Competing Interests statement in your cover letter; we will change the online submission form on your behalf.	updating the Competing Interests statement in the online submission form with this information.<br><br>Please also update the financial disclosure statement to read: “This research was supported by infrastructure funding from the HMRI Equity in Health and Wellbeing Research Program and an Australian Government Research Training (RTP) Scholarship”. This updated statement is also noted in the cover letter.
3. Please review your reference list to ensure that it is complete and correct. If you have cited papers that have been retracted, please include the rationale for doing so in the manuscript text, or remove these references and replace them with relevant current references. Any changes to the reference list should be mentioned in the rebuttal letter that accompanies your revised manuscript. If you need to cite a retracted article, indicate the article’s retracted status in the References list and also include a citation and full reference for the retraction notice.	Thank you for this advice. We have reviewed all references and updated reference citations and links accordingly, including the removal of a duplicated reference (original reference #18) and subsequent renumbering. We have also made some minor additions to text (lines 660-664 of tracked manuscript) due to information identified in a new reference.<br>“~~In Australia,~~ A 2022 national survey of trainee doctors in Australia found that 17% of IMG trainees and ~~report on Aboriginal and/or Torres Strait Islander doctors in training identified that~~ 39% of Aboriginal and/or Torres Strait Islander trainees had personally experienced bullying, harassment, discrimination and/or racism in the previous year, and that almost half (40% and 49% respectively) attributed ~~this to~~ the source as senior medical staff…
**Reviewer Comments to the Author**	
**Reviewer #1:**	
a) disadvantage is different from discrimination- explain how these two terms differ in your study.	Thank you for your interest in this important aspect of our research. Questions related to both ‘disadvantage’ and ‘discrimination’ were asked in the survey. A definition for ‘discrimination’ was provided for survey participants. A definition for ‘disadvantage’ was not provided in the survey and therefore left for individual interpretation of participants. Participant responses to both ‘disadvantage’ and ‘discrimination’ were explored in detail within the qualitative interviews, a benefit of the study being conducted with a sequential explanatory mixed methods approach. Our first qualitative theme: “Disadvantage as an independent construct to discrimination” explains the differences between

	'disadvantage' and 'discrimination', as reported by participants. This is further addressed in the 'Triangulation of findings' see lines 608-617
b) Be more clear in discussion on using social networks and snowball sampling may not fully represent all IMGs.	Thank you for this comment. We have now expanded the Limitation section to explicitly mention these recruitment paths. See lines 726-727 of tracked manuscript: "…the methods of recruitment (e.g., via social networks and snowball sampling) introduce potential bias to the study. Therefore, the ~~use of a~~ convenience sample may limit the generalizability of the findings, as it may not fully represent the broader IMG population
c) Add a short reflection that why senior IMGs bully others could be cultural hierarchies, internalized discrimination.	Thank you for this comment. Within the Results section (lines 540-543) our participants provided their own reflections about the underlying reasons why senior IMGs might bully: "Many theories of this phenomenon were postulated by interviewees, including subconscious cultural reactions, reasserting a familiar hierarchical relationship, internalised racism, power play and simply an opportunity to 'get away with it'." As this is an area not previously studied, we have made clearer reference to this in Discussion, to assist readers and future researchers. We have now included the following line in Discussion: "Theories postulated by our study participants (e.g., internal racism and cultural hierarchies) warrant ~~and therefore warrants~~ further exploration in future research." [lines 672-674 of tracked manuscript]
d) your recommendations are too general be more specific like mentoring program , support for exam preparation.	Thank you for this suggestion. We agree that more specific recommendations would be helpful for future practice, and have therefore made clearer mention of strategies plus the relevant institutions of interest, in lines 688-709 of tracked manuscript.<br>"This research supports previously postulated ~~solutions~~ recommendations to IMG inequity [11, 42]. Long-term, strategic approaches from institutions are needed to embed change and foster a culture of shared responsibility and accountability toward inclusivity and fair practice [43, 44]. Our results indicate that current institutional structures and policies in Australia which involve IMG assessment, regulatory licencing, employment, workplaces, training and immigration would benefit from review and change in order to ensure fairness and promotion of equitable practice.<br>Disadvantages experienced by IMGs as newcomers to a foreign work environment may be mitigated by experience sharing programs such as

	mentoring and peer support programs [11, 42]. Such programs may assist by providing a safe forum for IMGs to openly discuss challenges navigating new workplaces, expectations and roles [11,42]. Other recommendations include practical workplace assistance, such as support for examinations, training and language/communication courses. This is particularly relevant for those IMGs located in isolated geographical regions where inequities to training support are greater [11]. Our results indicate that IMGs may benefit from communication skills courses, to bridge written and spoken language deficits for IMGs, thereby improving opportunities at interviews and examination performance; whilst also benefitting communication with other staff and patients. ~~Such as experience sharing (e.g., support through mentoring and networking), workplace assistance (e.g. support for training and integration), structural review and change (e.g., removal of institutional barriers and promotion of fairness) and~~ Institutions have a vital role in fostering an equitable and inclusive environment, thereby promoting a culture of ~~promotion of~~ workplace harmony [11]. ~~(e.g., diversity training and fostering inclusion) [11]. Such solutions require long term, strategic approaches form organisations, in order to embed change and foster a culture of shared responsibility and accountability toward inclusivity [43,44].”~~
e) page 18 about cultural behaviors. Please simplify complex sentences they too long difficult to follow.	Thank you for this insight. We have now re-worded those sentences to make sentences easier to follow for the reader. Please see lines 394-398 in tracked version.<br>One interviewee from Trinidad and Tobago ~~reported~~ explained that their relaxed and ‘stress-free’ demeanour was misinterpreted by senior staff as disinterest in work. ~~Of “being [always relaxed, never sweat, never show stresss….all worked against my favour.~~ The interviewee continued to explain that ~~And~~ “…[my relaxed demeanour]…. worked against my favour. And that’s just how I am, because of where I’m from….”
f) And make sure every figure and table is mentioned where it is relevant in the text.	Thank you for this advice. We have reviewed the position of all supplemental material and adjusted signposting of these throughout the text; Lines: 156, 279, 287, 305, 311, 324- 325, 331 (retraction), 333-334, 355, 607, 617, 634, 640, 683, 721, 722 (in the tracked version)
**Reviewer #2:**	

This study highlights the widespread inequities and discrimination faced by International Medical Graduates (IMGs) in Australia, particularly emphasizing the effects of racial biases related to foreign degrees, language, and ethnicity. The sample size is small, which limits the generalizability of the findings. The use of both quantitative and qualitative methods enhanced the analysis, revealing important patterns and encouraging further investigation in future studies. While the results are promising, the exploratory nature of the research and the use of a convenience sample present limitations. Given the lack of comparable studies in this area, these findings provide a foundation for future research, promoting a deeper understanding of these issues.	Thank you for your positive and encouraging comments about our research. As identified by the reviewer, this study was primarily designed to be exploratory in nature, given the lack of similar studies existing in the current literature. We agree that sample size and convenience sampling poses limitations to interpretation and generalisability of our findings, and these are already noted in the Limitations section of the tracked version (lines 722-737).
