# Supplementary material for: Exploring experiences of work-related inequitable treatment among international medical graduates (IMGs): A sequential explanatory mixed methods study
Source: PLoS One. 2025 Feb 21;20(2):e0319230. doi: 10.1371/journal.pone.0319230 (PMC11845036; doi:10.1371/journal.pone.0319230)
Supplement: Attachment — Submitted filename: Response_to_reviewers_auresp_2.pdf [file pone.0319230.s012.pdf]

RESPONSE TO REVIEWERS

Dear Editor and Reviewers,

Re: PONE-D-24-43854: Exploring experiences of work-related inequitable treatment among international medical graduates (IMGs): a sequential explanatory mixed methods study

Thank you very much for your time and efforts in reviewing this manuscript. The authors are grateful for the many positive and encouraging comments made by the reviewers, and also for the insight and suggestions put forward. Please see our author responses below.

Kind regards, Dr S.J. Rebecca Healey and co-authors. 11/01/2025

Comment or request by Editor/Reviewer	Response from authors
**Academic Editor: Journal requirements**	
Please review your reference list to ensure that it is complete and correct.	Thank you for this advice. References have been checked and updated accordingly. Please note the following changes have been made:<br>1. In-text changes to reflect new up-to-date data from the longitudinal study referenced in Reference [38]- see Discussion lines 670-673. Reference [38] has also been updated accordingly within the Reference List<br>2. Addditional/updated information to Reference list pertaining to References [1], [18] and [20]<br>3. Three new in-text citations labelled [43], [44] and [47] with Reference added to the Reference list:<br><br>"43.*Workers rights and visa reporting protections [Internet]. Department of Home Affairs: Australian Government; 2024. [Cited 2025 Jan 16]. Available from: https://immi.homeaffairs.gov.au/visas/working-in-australia/work-rights-and-exploitation*"

	"44.Visa holders and migrants [Internet]. Fair Work Ombudsman: Australian Government. [Cited 2025 Jan 16]. Available from: https://www.fairwork.gov.au/find-help-for/visa-holders-migrants#our-role-as-the-fair-work-ombudsman"<br><br>*"47. Lee TC, McKinley SK, Dream SY, Grubbs EG, Dissanaike S, Fong ZV. Pearls and pitfalls of the virtual interview: perspectives from both sides of the camera. Journal of Surgical Research. 2021 Jun 1;262:240-3."*<br><br>4. Renumbering of References and citations within the document to account for three new in-text citations
**Reviewer comments to author**	
**Reviewer #3:** (No Response)	Thank you for your time and effort in reviewing our manuscript.
**Reviewer #4:** Overall, this is a very well-written article. It provides an excellent case for the need for a new study and research question, where there is a paucity of research. Abstract was clearly presented, with pertinent and was easy to read. The introduction aptly discussed prior research in this topic and briefly mentioned their results. It also describes the importance of IMGs to foreign economies, and the unique challenges they face to fulfill that need. Methods have been well-discussed, with good use of tables and figures. Authors can consider placing some tables and figures in the body of the article rather than supplemental material, as it may make it easier to follow visually. Results and discussion section is thorough and describes the nuances of the results. It also discusses the biases that may be present in the survey as compared to the interview method used. Limitations and strengths of the study are well-described. Compliments to the authors for presenting	Thank you for the positive comments made about our research and manuscript. Tables and figures have been placed as supplemental material in order to fulfil Journal requirements.

their information in a clear, concise way that is easy to read and is thought-provoking for further research.	
**Reviewer #5:** Extremely informative article, and nice to see more studies popping up about IMG disparities in multiple countries. Please see attached PDF file with all edits and comments for the article.	Thank you for the positive comments within the PDF file. Edits/suggestions made by Reviewer #5 within the PDF file are addressed by the authors below.
Minor editing/rephrasing suggestions in the PDF	The authors agree that the following minor rephrasing suggestions can be made throughout the document to improve readability in the following lines:<br>39: insertion of '*major*'<br>40: insertion of '*on this issue*'<br>94: replacement of 'migrant' with '*immigration*'<br>99: replacement of 'impactor' with '*impact*'<br>149: replacement of 'to participate' with '*in participating*'<br>150: insertion of '*the*'<br>155: replacement of 'seek' with '*gather*'<br>156: removal of 'which was'<br>195: pluralisation of 'report' to '*reports*'<br>201: replacement of 'Philippine' with '*Filipino*'<br>202: replacement of 'separate' with '*distinct*'<br>202: replacement of 'offered' with '*provided as an option*'<br>203: replacement of 'acknowledging' with '*reflecting*'<br>203: insertion of '*significant*'<br>207: replacement of 'system' with '*systemic*'<br>222: insertion of '*the*'<br>223-227: rephrasing of "Once themes where finalised, the research team compared the themes identified from both the survey open-ended responses and the interviews, reviewed for congruence and incorporated themes were able' to '*After finalising the themes, the research team conducted a comparative analysis of the themes derived from the survey open-ended*

	responses and interview data. They evaluated the alignment between the two source and integrated the themes where appropriate.'<br>232: insertion of '*had'*<br>264 and 281: replacement of 'Anaesthetics' with '*Anaesthesia*'<br>267: replacement of 'work-types' with '*work-settings*'<br>269: replacement of 'largely' with 'primarily'<br>269-271: rephrasing of '..thereby accounting for the similar demographic details of survey and interview participants observed in S3 Table' to '*…resulting in comparable demographic characteristics between survey and interview participants, as detailed in S3 Table.'*<br>308: insertion of commas between '…religion, name, age, sexual orientation…'<br>320: pluralisation of 'experience' to 'experiences'<br>324 and 325: replacement of 'to' with '*regarding'*<br>358-360: rephrasing of 'The primary discrimination tool (perceived discrimination in the last five years) was not statistically significantly associated with 'age'…' to '*The primary measure of perceived discrimination within the last five years demonstrated no statistically significant association with age…*'<br>394: replacement of 'in' with '*during'*<br>424-425: replacement of 'Also reported were…' with '*Other reports included*…'<br>487: replacement of 'told' with *'mentioned'*<br>489: replacement of IMG with '*IMG's'*<br>489: replacement of 'tiredness' with '*fatigue'*<br>492: insertion of '*to'*<br>513: replacement of 'told' with '*reported'*<br>579: rephrasing of 'complaints procedures' to '*the procedure to voice complaints*'<br>665: rephrasing of 'modern day' to '*the modern-day'*<br>739-740: replacement of 'were not able to' with '*could not be'*

	Please note, regarding line 451: the terminology 'thrice' was not changed to 'triple' as this was wording quoted by a participant.
[Referring to REDCAP] When using a data tool for a study , Please include information regarding the ease of Use, Data Collection Methods, Data Security and Privacy, Scalability, Analytical Capabilities, Ethics and Informed Consent, Reliability and Validity, Other strengths or limitations of the tool.	Thank you for your suggestion. Redcap is a frequently used tool for health research data collection and management. We have added some extra information about the REDCap tool under Data Collection [lines 175-180], with some rephrasing, such that these sentences now read:<br><br>*Data was collected, stored and managed using the REDCap (Research Electronic Data Capture) software platform, hosted through the Hunter Medical Research Institute [27, 28]. REDCap is an online, secure, data capture platform used in health research, providing an intuitive interface for data management and interoperability with common statistical packages [27, 28].*<br><br>Further details about the REDCap tool may be retrieved from the references already included in the manuscript or by visiting the REDCap website: https://projectredcap.org/software/
Was the online invitation an email link or a software/portal? Please do explain	Thank you for seeking clarification about the online invitation. The online invitation link was created through REDCap. The link was forwarded by electronic methods to participants e.g., email, social media post or newsletter. Clearer explanation has now been included in 'Data Collection' section [lines 180-183].
Was there the possibility of a potential bias since most were only interviewed virtually compared to in person ?	Thank you for raising this concept. Although the virtual interview format reduced participation bias for our study, we agree that other risks to bias may have been introduced. We have now made mention of this in the Limitations [lines 734-737], and included an additional reference [47].
Could you also include here what respondents mainly reported as the reason for IMGs being slightly/very advantaged compared to local graduates ? It would be interesting to point this out in the article.	Thank you for your interest in the findings from this part of the research. We have now included this data to the Quantitative Results section [lines 296-298] for interested readers.<br>Of the few (nine) participants who reported that IMGs were advantaged, the most common reasons were registration/bureaucracy (4/9) and assessment

	requirements (4/9). Unfortunately, we did not have the capacity to explore why these 9 participants reported favourably about these aspects (survey was anonymous), however further research exploring positive experiences would be beneficial.
[*Referring to the Results section: "A third of respondents (9/25;36%) identifying as British/Irish ethnicity reported discrimination in the last 5 years, compared to half (13/23; 56.52%) European/Eastern European respondents, and two thirds (91/140; 65%) respondents from all other ethnicities; p= 0.023"]* Is there a reason or explanation that can be provided for this difference based on ethnicity/race ?	Thank you for your query. Deeper explanation of these findings were sought in the qualitative component of the study and are described in our qualitative results section. Although we found multi-faceted explanations for this quantitative finding, it is concerning that most reasons point to racial biases (as mentioned in Discussion). Further exploratory research is indeed warranted in the area.
Do add or just mention any resources available to the article regarding IMG's workplace rights in Australia, to enhance further reading, which would make the article an even more interesting and informative read.	Thank you for this advice to enhance the manuscript. Australian workplace laws protect all workers (irrespective of visa or citizenship status), and all workers (including IMGs) may contact the Fair Work Ombudsman to receive free advice about workplace concerns. We have now included two sentences explaining this in the Discussion [lines 694-697] and include two new references numbered [43] and [44].
