# Supplementary material for: Exploring experiences of work-related inequitable treatment among international medical graduates (IMGs): A sequential explanatory mixed methods study
Source: PLoS One. 2025 Feb 21;20(2):e0319230. doi: 10.1371/journal.pone.0319230 (PMC11845036; doi:10.1371/journal.pone.0319230)
Supplement: Attachment — Submitted filename: Note to Editor.docx [file pone.0319230.s009.docx]

Dear Editor,

Thank you for seeking clarification about: “The individual Professor Kichu Nair named as investigator on the ethics documents is not an author on the present submission. As we would generally expect the investigator named on the ethical approval to be an author on the submission, can you please provide an explanation for this situation?”

Professor Kichu Nair is the same person as Balakrishnan R Nair. ‘Kichu’ is the shortened first name. Please see these websites: <https://www.profkichunair.com> and <https://www.newcastle.edu.au/profile/kichu-nair>. He is the Supervisory lead for the project. Therefore, his name was required for the Ethics submission.

Please advise if you need any further evidence to proceed with the submission.

Thank you,

Dr Healey
